# The association between parenting style and children’s loneliness after the COVID-19 pandemic: the mediating role of meaning in life

**DOI:** 10.3389/fpubh.2025.1688824

**Published:** 2026-02-11

**Authors:** Qi Yang, Zhe Jiang, Hongtu Xu, Gengfeng Niu, Humin Yang, Huan Li

**Affiliations:** 1 College of Education, Fuyang Normal University, Fuyang, China; 2 School of Education and Science, Huangshan University, Huangshan, China; 3 Key Laboratory of Adolescent Cyberpsychology and Behavior (CCNU), Ministry of Education, Wuhan, China; 4 Key Laboratory of Human Development and Mental Health of Hubei Province, School of Psychology, Central China Normal University, Wuhan, China; 5 School of Educational Science, Anhui Normal University, Wuhu, China

**Keywords:** children, loneliness, meaning in life, mediation, parenting style, the post-COVID-19 pandemic

## Abstract

Strict lockdown measures during the COVID-19 pandemic altered patterns of social interaction, leading to increased social isolation and heightened levels of loneliness in the post-pandemic era. However, it remains unclear whether children continue to experience elevated loneliness after the pandemic’s official end. Loneliness represents a prominent negative emotional state among children, is associated with various adverse outcomes, and warrants particular attention in the current post-pandemic context. Parenting constitutes a key familial factor that profoundly influences children’s psychological development and social adaptation. Grounded in the Social, Economic, and Cultural Framework and the Distal-Structural-Proximal Model, this study examined the association between parenting style and children’s loneliness after the COVID-19 pandemic, as well as the potential mediating role of meaning in life. A total of 446 elementary school students participated in the study on 16 June 2023. The results indicated that the children’s level of loneliness was below the medium level and that loneliness was negatively associated with positive parenting and positively associated with negative parenting. Moreover, meaning in life significantly mediated the relationship between positive parenting and loneliness but not between negative parenting and loneliness. These findings advance our understanding of the complex interplay among parenting, meaning in life, and loneliness and offer practical implications for parenting interventions aimed at supporting children’s psychological adjustment in the post-pandemic era.

## 1 Introduction

Although public health restrictions related to the COVID-19 pandemic have been lifted globally, its enduring impacts on individuals’ social adaptation persist—including elevated levels of loneliness (1–3). Loneliness refers to a subjective experience wherein individuals feel dissatisfied with their social relationship networks, typically accompanied by negative emotional reactions, such as sadness and emptiness (4). Although it is a common experience across age groups, children are particularly susceptible to experiencing loneliness at school (4). Meanwhile, loneliness has a wide influence on children’s health and wellbeing (5), and it is found to be closely related to behavioral problems (6, 7), academic difficulties (8), social barriers (9, 10), and even depression (11, 12). Consequently, children’s loneliness has garnered substantial attention, especially during and after the COVID-19 pandemic (4, 13). Moreover, individuals’ social interaction styles were altered due to the strict lockdown during the COVID-19 pandemic, leading to increased social isolation and, consequently, higher levels of loneliness (14). Based on these views, the present study aims to examine the underlying mechanism of children’s loneliness after the COVID-19 pandemic.

Numerous studies have identified that family-related factors [e.g., family financial status (15); parent–child attachment (15); and parenting practices (16)] play a key role in children’s social adaptation and development. Among these factors, parenting style is particularly important and is defined as the consistent approach parents employ in raising their children (17). It can be divided into positive and negative parenting. As we know, the COVID-19 pandemic not only exerted adverse effects on children’s mental health but also affected parents’ daily routines, thereby impacting their parenting practices (18). Parenting style lays an important foundation for the mental health of children. For example, positive parenting fosters a supportive environment, conducive to children’s emotional wellbeing (19), self-esteem (20), and social skills (21); in contrast, negative parenting often involves harmful or ineffective practices that can lead to adverse physical and mental health outcomes in children (16, 22, 23). Regarding loneliness, positive parenting (e.g., warmth) has been negatively associated with loneliness among primary school students (24), while negative parenting (e.g., over-protective and inhibitive behaviors) has been positively associated with loneliness (25, 26). Based on the aforementioned research findings, the present study hypothesizes that negative parenting is positively associated with children’s loneliness, while positive parenting is negatively associated with children’s loneliness.

### 1.1 The mediating role of meaning in life

Meaning in life refers to the subjective sense of purpose, significance, and fulfillment that individuals perceive in their existence (27). It is a key predictor of mental health and wellbeing across populations (28), especially for children. Examining children’s meaning in life in the post-COVID-19 pandemic thus contributes to a deeper understanding of their mental health.

Regarding its relation to loneliness, meaning in life emerges from social connections, which contrasts with the core experience of loneliness—unmet social needs (4, 29). Loneliness also involves a judgment of the meaning and adequacy of one’s social connections, which presupposes an individual’s cognitive framework of values and expectations. In other words, meaning in life is an important predictor of individual loneliness. Previous studies have demonstrated that meaning in life is closely and negatively associated with loneliness (30–33). Based on these findings, the present study proposes that meaning in life is negatively associated with feelings of loneliness. Furthermore, studies indicate that the COVID-19 pandemic has undermined individuals’ sense of meaning in life (34, 35), largely because lockdowns and physical distancing measures disrupted the social relationships through which children derive meaning. Cultivating a clear sense of meaning in life is therefore crucial for fostering children’s self-confidence and positive attitudes in the post-pandemic period.

Regarding the development of meaning in life, Fry (36) argued that individuals must cultivate both psychological assets (e.g., life skills for effective coping and adaptation to societal expectations) and social assets (e.g., peers and family) to attain meaning in life, among which parenting is an important element. Parents typically teach their children how to think and how to relate with other people and their environment, roughly corresponding to parenting behavior. For example, parental support, as a kind of positive parenting, reflects the extent to which an individual feels appreciated and encouraged by his or her parents (37), and it could foster a sense of meaning in life. Consequently, positive parenting is hypothesized to foster meaning in life, thereby reducing loneliness. In contrast, negative parenting may reduce meaning in life, ultimately increasing loneliness.

The *Distal-Structural-Proximal Model* (38) and the *Social, Economic, and Cultural Framework* (39) address the risk factors for loneliness (40) and provide theoretical support for the role of meaning in life as a mediator in the relationship between parenting style and loneliness. According to these frameworks, external factors (i.e., parenting style) influence loneliness through proximal individual factors (here, i.e., meaning in life). Given that meaning in life is a core personal resource consistently linked to mental health and psychological wellbeing across the lifespan (41) and that previous studies have identified meaning in life as a mediator between family/external environment factors and mental health (65, 66), the present study hypothesizes that meaning in life mediates the relationship between parenting style and children’s loneliness.

### 1.2 The present study

Given the enduring impacts of the COVID-19 pandemic on children’s mental and physical health, particularly in the post-pandemic era, the present study integrates relevant theoretical and empirical evidence to construct a mediating model. In this model, parenting style serves as the predictor variable, children’s loneliness as the outcome variable, and meaning in life as the mediating variable. The study aims to deepen understanding of the formation processes and influencing factors of children’s loneliness, with the ultimate goal of providing actionable insights to reduce loneliness among children in the post-COVID-19 period.

## 2 Method

### 2.1 Participants

A total of 477 students in grades 4 to 6 from a primary school in central China were recruited using a convenience sampling method on 16 June 2023. These participants were asked to complete a paper-and-pencil questionnaire. A total of 20 students whose responses to items showed no variation on at least one scale and one student who left more than 10% of the questions unanswered were removed from further analysis. This procedure resulted in the final sample size of 446 students (93.50%). Among them, there were 240 girls and 204 boys, with two participants not reporting gender. The sample included 142 6th-grade students, 161 5th-grade students, and 143 4th-grade students. Their average age was 11.77 ± 0.87 years. All students and their legal guardians provided written informed consent, and the study was approved by the ethics committee of the authors’ institution.

### 2.2 Measurements

#### 2.2.1 Children’s loneliness

The Chinese version (67) of the Children’s Loneliness Scale developed by Asher et al. (42) was used to assess the children’s level of loneliness. This scale contains 24 items, and the scores of items related to individual hobbies were excluded for assessing the level of loneliness. The participants were asked to respond on a five-point Likert scale ranging from 1 (never) to 5 (always), with higher average scores indicating greater perceived loneliness. In the present study, Cronbach’s alpha was 0.88.

#### 2.2.2 Parenting style

The Chinese version (68) of the Parenting Style Scale was used to assess positive and negative parenting. This scale contains 21 items, comprising a positive parenting subscale (7 items) and a negative parenting subscale (14 items). The participants were asked to respond on a four-point Likert scale ranging from 1 (never) to 4 (always), with higher average scores indicating greater levels of perceived positive or negative parenting. In the present study, Cronbach’s alpha for positive parenting was 0.84 and for negative parenting was 0.83.

#### 2.2.3 Meaning in life

The Chinese version (43) of the Meaning in Life Scale developed by Steger et al. (27) was used to assess the children’s perceived level of meaning in life. This scale contains 10 items. The participants were asked to respond on a seven-point Likert scale ranging from 1 (completely inconsistent) to 7 (completely consistent), with higher average scores indicating a greater perceived level of meaning in life. In the present study, Cronbach’s alpha was 0.81.

### 2.3 Procedure

Before the survey, the primary school administrator sought oral informed consent from all parents regarding their children’s participation in the study. Subsequently, paper-and-pencil questionnaires were distributed to all students in their classrooms by three university students majoring in psychology. The three students were trained before conducting this study. The students were given 45 min to complete the questionnaire and were encouraged to raise their hands if they encountered unfamiliar concepts or had any questions; the three university students were available to provide clarification as needed. Finally, their teachers collected all questionnaires.

### 2.4 Data analysis

SPSS 27.0 and the SPSS PROCESS macro,^1^ as recommended by Hayes were used to analyze the data. First, factor analysis was used to examine whether common method bias was a concern in the present study. Second, descriptive statistics, mean differences, and partial correlations were calculated. Subsequently, Hayes’s SPSS PROCESS macro (Model 4) was used to test the mediation model. Furthermore, we used 5,000 bias-corrected bootstrap resamples to estimate the 95% confidence interval (with intervals not containing zero indicating statistical significance) of the mediating effect.

## 3 Results

### 3.1 Common method bias test

As all the questionnaire data in this study were obtained via self-report from the participants, the present study may be affected by common method bias. We performed Harman’s single-factor test on all items from the three questionnaires (44). The results showed that there were 20 factors with eigenvalues greater than 1, and the variance explained by the first factor was only 15.75%, which is below the critical threshold of 40%. Therefore, it can be concluded that this study did not exhibit serious common method bias.

### 3.2 Descriptive statistics and correlational analysis

Means, standard deviations, and Pearson’s correlation coefficients are summarized in Table 1. The results of the correlation analysis showed that children’s loneliness was negatively associated with positive parenting (*r* = −0.40, *p* < 0.01) and positively associated with negative parenting (*r* = 0.23, *p* < 0.01) and meaning in life (*r* = −0.26, *p* < 0.01). Moreover, meaning in life was positively correlated only with positive parenting (*r* = 0.28, *p* < 0.01).

**Table 1:** Descriptive statistics and correlations among all variables (*N* = 446).

Variable	1	2	3	4	5	6
1. Gender	–					
2. Age	−0.01	–				
3. Children’s loneliness	0.06	−0.21**	–			
4. Meaning in life	−0.06	0.01	−0.26**	–		
5. Positive parenting	−0.04	0.06	−0.40**	0.28**	–	
6. Negative parenting	−0.04	−0.07	0.23**	−0.02	−0.10*	–
M	1.54	11.77	2.03	4.55	2.73	1.76
SD	0.50	0.87	0.75	1.19	0.67	0.37
Range	–	–	[1, 3.31]	[1.6, 5.55]	[1, 3.14]	[1, 2.87]
Kurtosis	–	–	−0.38	−0.31	−0.62	2.87

*p < 0.05; **p < 0.01. Gender was dummy coded as Gender was dummy coded as 1 (boy) and 2 (girl). The Kurtosis value indicates the distribution of the data surveyed.

### 3.3 Mediation model testing

The main results of the mediation analysis, generated using Hayes’s SPSS PROCESS macro, are presented in Table 2. As shown, after controlling for gender and age, positive parenting was negatively associated with children’s loneliness (*β* = −0.43, *p* < 0.001) and positively associated with meaning in life (*β* = 0.50, *p* < 0.001). As shown in the outcome variable model predicting meaning in life, after controlling for gender and age, positive parenting was negatively associated with children’s loneliness (*β* = −0.38, *p* < 0.001), and meaning in life was negatively associated with children’s loneliness (*β* = −0.10, *p* < 0.01).

**Table 2:** Testing the mediation effect of meaning in life after controlling for gender and age (*N* = 446).

Outcome variable	Predictors	*R*	*R* ^2^	*F*	*β*	*t*	95% CI
Loneliness	Positive PS	0.44	0.20	35.54	−0.43	−9.00^***^	[−0.53, −0.34]
Meaning in life	Positive PS	0.28	0.08	12.77	0.49	6.05^***^	[0.33, 0.65]
Loneliness	Positive PS	0.47	0.22	30.77	−0.38	−7.75***	[−0.48, −0.29]
	Meaning in life				−0.10	−3.66**	[−0.16, −0.05]

CI, confidence interval. Gender was dummy coded as 0.5 (boy) and −0.5 (girl). PS indicated parenting style. **p < 0.01; ***p < 0.001.

Next, a bootstrap procedure was used to further test and calculate the mediating effects found in our mediation model. It was observed that the 95% confidence intervals for the mediating paths did not include 0, indicating that the mediating effect was significant. The mediating effect value was −0.05; the mediating path accounted for 11.90% of the total effect of positive parenting on children’s loneliness, representing the ratio of the indirect effect to the total effect. The effect values for the mediating paths are presented in Table 3.

**Table 3:** Total, direct, and indirect effects of positive parenting style (*X*) on children’s loneliness (*Y*) through meaning in life (*M*) (*N* = 446).

Effect	Value	SE	LLCI	ULCI	Relative value
Total effect	−0.43	0.05	−0.53	−0.34	–
Direct effect	−0.38	0.05	−0.48	−0.29	–
Indirect effect	−0.05	0.02	−0.09	−0.02	11.63%

SE, standard error; CI, confidence interval; LL, low limit; UL, upper limit.

## 4 Discussion

In recent years, the lasting and adverse effects of the COVID-19 pandemic on children’s mental health and social adaptation have been documented in many studies (1–3). Moreover, children’s loneliness is a key indicator of their social development and adaptation in the post-pandemic period. Against this background, the present study focused on loneliness among Chinese children. Based on theoretical and empirical research, this study sought to examine the current status of children’s loneliness, with a particular emphasis on the mediating role of meaning in life in the complex relationship between parenting styles and children’s loneliness. The current investigation revealed that there was an inverse correlation between positive parenting and children’s loneliness, coupled with a positive association between negative parenting and children’s loneliness. Notably, meaning in life was identified as a partial mediator in the relationship between positive parenting and children’s reported loneliness but not in the relationship between negative parenting and children’s reported loneliness. These findings provide new insights into the understanding of children’s loneliness, particularly in the post-pandemic period.

### 4.1 The current status of children’s loneliness

Although previous research has demonstrated significant adverse effects of the COVID-19 pandemic on parents—particularly due to changes in work mode, lifestyle, and economic instability—the present study found relatively low levels of children’s loneliness (M = 2.03) during the post-pandemic period. This finding appears inconsistent with previous research (18). Nevertheless, this pattern of results may be explained by at least two factors. First, unlike the impact of the COVID-19 pandemic on parents, students who had quick access to peer support at school may have been better able to mitigate or buffer the negative psychological impacts of the pandemics particularly those stemming from altered social interactions. Second, students with higher levels of psychological resilience or greater access to internal and external psychological resources may have had the capacity to recover more effectively from pandemic-related mental health challenges. Moreover, Zdziarski et al. (45) also reported that adults generally provided positive assessments of students’ life satisfaction after the pandemic.

Admittedly, some might argue that this study has certain methodological limitations. For instance, because data were collected in a school setting, children’s responses could have been influenced by social desirability bias. To minimize this effect, we administered the questionnaire anonymously. Future research could further validate our findings by incorporating additional methodologies or converging evidence from diverse sources.

### 4.2 The association between parenting and children’s loneliness

Aligning with prior research (24–26), the current study showed a significant negative association between children’s loneliness and positive parenting and a positive association between children’s loneliness and negative parenting. Previous studies have also shown that parental warmth (a common form of positive parenting) is negatively related to children’s loneliness, whereas parental rejection (a common form of negative parenting) is positively related to children’s loneliness (24–26). These findings have also been observed among adolescents with divorced parents (46) and among left-behind children (47), indicating the importance of parenting for the mental health of children. Previous studies have also suggested that positive parenting, particularly those characterized by democratic approaches, plays a pivotal role in preventing or mitigating loneliness in primary school students, while negative parenting, such as authoritarian and permissive styles, is linked to an increased likelihood of loneliness (48). Our findings, combined with previous findings, are consistent with the perspective proposed by the risk factor models of loneliness—specifically, the *Distal-Structural-Proximal Model* (38) and the *Social, Economic, and Cultural Framework* (39). According to these two models, parenting—as an important sociocultural and distal factor—could be correlated with children’s loneliness. More precisely, parenting could reduce children’s loneliness by teaching them some interpersonal skills or by providing positive feedback that enhances their sense of psychological safety (49). Therefore, parents should carefully consider their parenting style, reduce negative parenting practices, and adopt more positive parenting approaches to reduce loneliness among primary school students in the post-COVID-19 period.

### 4.3 The mediating role of meaning in life

Consistent with empirical and theoretical perspectives, we further found that meaning in life partially mediated the relationship between positive parenting and children’s loneliness, while it did not mediate the relationship between negative parenting and loneliness.

First, this study found that positive parenting positively predicted meaning in life, while the impact of negative parenting on meaning in life was not significant. These findings are generally consistent with previous studies. Previous research has found that parental monitoring (50), authoritative parenting (51) family environment (52, 53), and social support (54) are correlated with meaning in life among adolescents. These studies indicate that positive parenting or social support has a positive association with the perception of meaning in life, whereas negative parenting or lack of social support exerts a negative impact on children’s sense of meaning in life (55). Positive parenting can enhance children’s sense of meaning in life through encouragement, support, and understanding (56). Negative parenting may be related to a lack of positive support, which can lead to greater feelings of helplessness and confusion (37).

However, we did not observe a significant association between negative parenting and meaning in life. Previous studies demonstrate that authoritative parenting has a very limited impact on individuals’ mental health (57). It may be because, even under negative parenting, a child’s sense of meaning in life can remain relatively high, as children may derive positive meaning through participation in external and social activities (58). This indicates that the impact of parenting style on meaning in life is not purely direct, and various factors, such as individual differences, can also influence this relationship. Therefore, the finding of the adverse effect of negative parenting on meaning in life should be confirmed in future studies. In summary, parenting style is closely and intricately associated with meaning in life.

Notably, data for this study were collected after the official end of the COVID-19 pandemic, during a period when societal life had largely returned to normal. In this post-pandemic phase, although children had resumed in-person schooling, their psychological adjustment was still ongoing. At this stage, positive parenting practices may help children ascribe meaning to their pandemic-related experiences (e.g., “We got through a difficult time together” or “You’ve become stronger because of this”), thereby fostering a sense of meaning in life and subsequently buffering feelings of loneliness. In contrast, the impact of negative parenting on loneliness may operate more directly through daily interactions characterized by emotional neglect or behavioral control, with its pathways primarily linked to emotional security or social competence rather than existential dimensions, such as whether life feels valuable or purposeful.

Second, meaning in life had a significant negative predictive effect on loneliness in primary school students, which aligns with previous studies. Considerable direct evidence shows that meaning in life is not only negatively related to loneliness (31–33, 59) but also serves as a protective factor in reducing children’s levels of loneliness (30, 31). Evidence from indirect studies has shown that positive mood and social relatedness are positively associated with meaning in life (59) and negatively correlated with feelings of loneliness (60). Although these findings were not obtained from children, they highlight the logical relationship between these factors. In particular, meaning in life mainly emerges from social interaction, and loneliness is generally related to the failure of social interpersonal relationships. Based on both empirical evidence and theoretical reasoning, meaning in life is proposed to be negatively associated with children’s loneliness.

To summarize, these findings are also consistent with the views of the *Distal-Structural-Proximal Model* (38) and the *Social, Economic, and Cultural Framework* (39). In addition, positive parenting may enhance children’s sense of meaning in life by providing them with warmth and support. Therefore, family factors, such as parenting style, as perceived by children, could exert an impact on children’s loneliness through proximal factors, such as meaning in life.

### 4.4 Implications and limitations

The current study contributes to the understanding of children’s loneliness by empirically scrutinizing the correlation between parenting styles and loneliness in primary school students, while also exploring the mediating role of meaning in life. Specifically, the mediating role of meaning in life was found to be associated exclusively with positive parenting and the manifestation of loneliness in children. Practically, the present study highlights the importance of both positive and negative parenting. More specifically, in the context of family education, reducing negative parenting practices and adopting more positive approaches may help decrease the level of loneliness in children. In addition, the study highlights the potential effectiveness of enhancing children’s sense of meaning in life as a way to alleviate loneliness, consistent with previous studies. For instance, Macià et al. (31) showed that meaning in life, compared to health status and social connectedness, was a more important predictor of loneliness. In fact, at the time of our survey, China’s pandemic control measures had already become considerably relaxed. For example, health QR codes were no longer required, and travel restrictions had been lifted. Moreover, a large proportion of the population had received COVID-19 vaccinations. This study provides valuable empirical evidence regarding the factors influencing children’s loneliness in the post-pandemic period. It also offers insights that may inform future responses to potential public health crises and their impact on primary school students’ mental well-being, particularly their experience of loneliness.

Nonetheless, several limitations warrant acknowledgment. First, although children were allowed to raise their hands during the questionnaire to seek clarification on abstract concepts or any other questions related to the scale items, some items in the Meaning in Life Scale may still have been too abstract for them to fully understand. Additional analysis revealed that the level of meaning in life did not differ significantly across grades [*F*(2, 443) = 0.787, *p* = 0.46]. Therefore, a child-friendly version of the scale should be used in future studies. Second, social desirability bias may have influenced the responses of children regarding their parents’ parenting styles. In such situations, children may be reluctant to evaluate their parents negatively. To address this concern, children were allowed to complete the questionnaire anonymously. Moreover, we observed a similar response pattern, with positive items receiving higher ratings than negative items on the Parenting Style Scale among Chinese children (61, 62). In addition, if we had asked the parents to complete the parenting styles questionnaire, we might have observed an even greater social desirability bias in their responses. Third, the cross-sectional design of this study precludes the establishment of causal inferences; therefore, longitudinal or follow-up studies are recommended. Fourth, existing research suggests that children’s loneliness is influenced by an array of additional factors (63), such as other family environmental factors and family socioeconomic status (64). Future research should systematically investigate the interplay between these variables. Moreover, all participants were drawn from a single primary school in central China, where sociocultural homogeneity, as well as socioeconomic and regional factors, may limit the generalizability of the findings. Finally, although this study was conducted in the post-pandemic period, the lack of empirical data from the COVID-19 pandemic prevents a direct comparison of meaning in life and loneliness among children between the COVID-19 pandemic and post-COVID-19 pandemic periods. Future study could examine this issue using a meta-analysis approach.

## Data Availability

The raw data supporting the conclusions of this article will be made available by the authors, without undue reservation.

## Footnotes

1
http://www.afhayes.com

## References

[1] Ernst M Niederer D Werner AM Czaja SJ Mikton C Ong AD . Loneliness before and during the COVID-19 pandemic: a systematic review with Meta-analysis. Am Psychol. (2022) 77:660–77. doi: 10.1037/amp0001005, 35533109 PMC9768682

[2] Houghton S Kyron M Hunter SC Lawrence D Hattie J Carroll A . Adolescents’ longitudinal trajectories of mental health and loneliness: the impact of COVID-19 school closures. J Adolesc. (2022) 94:191–205. doi: 10.1002/jad.12017, 35353417 PMC9087620

[3] Schneider V Norris T Nugawela M Dalrymple E Hargreaves D Käll A . Loneliness trajectories, associated factors and subsequent health in children and young people during the COVID-19 pandemic: a national matched cohort study. Psychol Res Behav Manag. (2023) 16:4461–77. doi: 10.2147/PRBM.S421165, 37936971 PMC10626032

[4] Asher SR Paquette JA. Loneliness and peer relations in childhood. Curr Dir Psychol Sci. (2003) 12:75–8. doi: 10.1111/1467-8721.01233

[5] Holt-Lunstad J Smith TB Baker M Harris T Stephenson D. Loneliness and social isolation as risk factors for mortality: a meta-analytic review. Perspect Psychol Sci. (2015) 10:227–37. doi: 10.1177/1745691614568352, 25910392

[6] McKay MT Konowalczyk S Andretta JR Cole JC. The direct and indirect effect of loneliness on the development of adolescent alcohol use in the United Kingdom. Addict Behav Rep. (2017) 6:65–70. doi: 10.1016/j.abrep.2017.07.003, 29450238 PMC5800553

[7] Zach S Yazdi-Ugav O Zeev A. Academic achievements, behavioral problems, and loneliness as predictors of social skills among students with and without learning disorders. School Psychol Int. (2016) 37:378–96. doi: 10.1177/0143034316649231

[8] Benner AD. Latino adolescents’ loneliness, academic performance, and the buffering nature of friendships. J Youth Adolesc. (2011) 40:556–67. doi: 10.1007/s10964-010-9561-2, 20571900 PMC3033456

[9] Goll JC Charlesworth G Scior K Stott J. Barriers to social participation among lonely older adults: the influence of social fears and identity. PLoS One. (2015) 10:e0116664. doi: 10.1371/journal.pone.0116664, 25706933 PMC4338142

[10] Tan M Shallis A Barkus E. Social anhedonia and social functioning: loneliness as a mediator. PsyCh J. (2020) 9:280–9. doi: 10.1002/pchj.344, 31965741

[11] Kılınç G Aylaz R Güneş G Harmancı P. The relationship between depression and loneliness levels of the students at the faculty of health sciences and the factors affecting them. Perspect Psychiatr Care. (2020) 56:431–8. doi: 10.1111/ppc.12452, 31721230

[12] Wang S Quan L Chavarro JE Slopen N Kubzansky LD Koenen KC . Associations of depression, anxiety, worry, perceived stress, and loneliness prior to infection with risk of post–COVID-19 conditions. JAMA Psychiatry. (2022) 79:1081–91. doi: 10.1001/jamapsychiatry.2022.2640, 36069885 PMC9453634

[13] Farrell AH Vitoroulis I Eriksson M Vaillancourt T. Loneliness and well-being in children and adolescents during the COVID-19 pandemic: a systematic review. Children. (2023) 10:279. doi: 10.3390/children10020279, 36832408 PMC9955087

[14] Zeng Y Song J Zhang Y Guo X Xu X Fan L . Life changes and symptoms of depression and anxiety among Chinese children and adolescents before, during, and after the COVID-19 pandemic lockdown: a combination of cross-sectional, longitudinal, and clustering studies. Eur Child Adolesc Psychiatry. (2025) 34:1025–38. doi: 10.1007/s00787-024-02533-4, 39060517

[15] Yang Q Zhang W Wu H Huang B Zhang C Niu G. The association between perceived family financial stress and adolescent suicide ideation: a moderated mediation model. Behav Sci. (2023) 13:948. doi: 10.3390/bs13110948, 37998693 PMC10669165

[16] Flannery AJ Awada SR Shelleby EC. Influences of maternal parenting stress on child behavior problems: examining harsh and positive parenting as mediators. J Fam Issues. (2023) 44:1215–36. doi: 10.1177/0192513X211056207

[17] Baumrind D. The influence of parenting style on adolescent competence and substance use. J Early Adolesc. (1991) 11:56–95. doi: 10.1177/0272431691111004

[18] Rodriguez V Cottrell J Jia F. Parental stress, parent–child relationship, and child wellbeing: a national study of family life after COVID-19 pandemic. Behav Sci. (2025) 15:1423. doi: 10.3390/bs15101423, 41153212 PMC12561007

[19] Santos-Nunes M Narciso I Vieira-Santos S Roberto MS. Parenting and emotional well-being of adoptive school-aged children: the mediating role of attachment. Child Youth Serv Rev. (2017) 81:390–9. doi: 10.1016/j.childyouth.2017.08.026

[20] Pinquart M Gerke DC. Associations of parenting styles with self-esteem in children and adolescents: a meta-analysis. J Child Fam Stud. (2019) 28:2017–35. doi: 10.1007/s10826-019-01417-5

[21] Jeon S Neppl TK. Economic pressure, parent positivity, positive parenting, and child social competence. J Child Fam Stud. (2019) 28:1402–12. doi: 10.1007/s10826-019-01372-1

[22] Goagoses N Bolz T Eilts J Schipper N Schütz J Rademacher A . Parenting dimensions/styles and emotion dysregulation in childhood and adolescence: a systematic review and meta-analysis. Curr Psychol. (2023) 42:18798–822. doi: 10.1007/s12144-022-03037-7

[23] O’Brien JR Loi EC Byrne ML Zalewski M Casement MD. The link between positive and negative parenting behaviors and child inflammation: a systematic review. Child Psychiatry Hum Dev. (2023) 54:51–65. doi: 10.1007/s10578-021-01224-4, 34347228 PMC8814056

[24] Yang X Fan C Liu Q Chu X Song Y Zhou Z. Parenting styles and children’s sleep quality: examining the mediating roles of mindfulness and loneliness. Child Youth Serv Rev. (2020) 114:104921. doi: 10.1016/j.childyouth.2020.104921

[25] Jackson T. Protective self-presentation, sources of socialization, and loneliness among Australian adolescents and young adults. Pers Individ Differ. (2007) 43:1552–62. doi: 10.1016/j.paid.2007.04.012

[26] Türkmen M Demirli A. The predictive value of gender, perceived parenting styles and loneliness in determining students’ dispositional and state hope level. J Theory Pract Educ. (2011) 7:347–63.

[27] Steger MF Frazier P Oishi S Kaler M. The meaning in life questionnaire: assessing the presence of and search for meaning in life. J Counsel Psychol. (2006) 53:80. doi: 10.1037/0022-0167.53.1.80

[28] Lin L Chan HW. When is search for meaning in life beneficial for well-being? A cross-national study. Int J Psychol. (2021) 56:75–84. doi: 10.1002/ijop.12696, 32596852

[29] Cipolletta S Ronconi L Tomaino SCM. Social connections combat loneliness and promote wellbeing among college students coming out of the COVID-19 pandemic. Front Psychol. (2025) 16:1529795. doi: 10.3389/fpsyg.2025.1529795, 40045975 PMC11879988

[30] Folker AP Lauridsen SM Hegelund ER Wimmelmann CL Flensborg-Madsen T. Does meaning protect against loneliness? Exploring empirical studies and theory. Health Promot Int. (2021) 36:471–80. doi: 10.1093/heapro/daaa081, 32830234

[31] Macià D Cattaneo G Solana J Tormos JM Pascual-Leone A Bartrés-Faz D. Meaning in life: a major predictive factor for loneliness comparable to health status and social connectedness. Front Psychol. (2021) 12:627547. doi: 10.3389/fpsyg.2021.627547, 33716892 PMC7943478

[32] Ozawa-de Silva C. In the eyes of others: loneliness and relational meaning in life among Japanese college students. Transcult Psychiatry. (2020) 57:623–34. doi: 10.1177/1363461519899757, 32041496

[33] Yıldırım M Arslan G Wong PT. Meaningful living, resilience, affective balance, and psychological health problems among Turkish young adults during coronavirus pandemic. Curr Psychol. (2021) 41:7812–23. doi: 10.1007/s12144-020-01244-8, 33424205 PMC7785475

[34] Baños RM Desdentado L Vara MD Escrivá-Martínez T Herrero R Miragall M . How the COVID-19 pandemic and its consequences affect the presence of and search for meaning of life: a longitudinal study. J Happiness Stud. (2022) 24:17–33. doi: 10.1007/s10902-022-00592-5, 36312909 PMC9595083

[35] Kumar V Sharma N. Role of COVID-19 obsession and death anxiety on quality of life of Indian population. Ind J Health Wellbeing. (2023) 14:365–8.

[36] Fry PS. The development of personal meaning and wisdom in adolescence: a reexamination of moderating and consolidating factors and influences. New Jersey: Lawrence Erlbaum Associates Publishers (1998).

[37] Barber BK Stolz HE Olsen JA. Parental support, psychological control, and behavioral control: assessing relevance across time, method, and culture. Monogr Soc Res Child Dev. (2005) 70:73–95. doi: 10.1111/j.1540-5834.2005.00370.x, 16359423

[38] Hawkley LC Hughes ME Waite LJ Masi CM Thisted RA Cacioppo JT. From social structural factors to perceptions of relationship quality and loneliness: the Chicago health, aging, and social relations study. J Gerontol Ser B Psychol Sci Soc Sci. (2008) 63:S375–84. doi: 10.1093/geronb/63.6.S375, 19092047 PMC2769562

[39] de Jong Gierveld J van Tilburg TG Dykstra PA. Loneliness and social isolation In: Perlman D Vangelisti A, editors. The Cambridge handbook of personal relationships. Cambridge: Cambridge University Press (2006). 485–500.

[40] Barjaková M Garnero A d’Hombres B. Risk factors for loneliness: a literature review. Soc Sci Med. (2023) 334:116163. doi: 10.1016/j.socscimed.2023.116163, 37625251 PMC10523154

[41] Steger MF Oishi S Kashdan TB. Meaning in life across the life span: levels and correlates of meaning in life from emerging adulthood to older adulthood. J Posit Psychol. (2009) 4:43–52. doi: 10.1080/17439760802303127

[42] Asher SR Hymel S Renshaw PD. Loneliness in children. Child Dev. (1984) 55:1456–64. doi: 10.2307/1130015

[43] Wang X. Psychometric evaluation of the meaning in life questionnaire in Chinese middle school students. Chin J Clin Psychol. (2013) 21:764–7.

[44] Livingstone LP Nelson DL Barr SH. Person-environment fit and creativity: an examination of supply-value and demand-ability versions of fit. J Manag. (1997) 23:119–46. doi: 10.1177/014920639702300202

[45] Zdziarski K Knyszyńska A Karakiewicz-Krawczyk K Awad M Awad S Qumsieh N . Life satisfaction of Palestinian and polish students after pandemic COVID-19. Front Public Health. (2025) 12:1409710. doi: 10.3389/fpubh.2024.1409710, 39944567 PMC11813783

[46] Lan X. Disengaged and highly harsh? Perceived parenting profiles, narcissism, and loneliness among adolescents from divorced families. Pers Individ Differ. (2021) 171:110466. doi: 10.1016/j.paid.2020.110466

[47] Song J Ma C Ruan Y. Left-behind children's grandparent–child and parent–child relationships and loneliness: a multivariable mediation model. Fam Relat. (2021) 70:195–206. doi: 10.1111/fare.12480

[48] Lim S Smith J. The structural relationships of parenting style, creative personality, and loneliness. Creat Res J. (2008) 20:412–9. doi: 10.1080/10400410802391868

[49] Fan J Zhang LF. The role of perceived parenting styles in thinking styles. Learn Individ Differ. (2014) 32:204–11. doi: 10.1016/j.lindif.2014.03.004

[50] Malinakova K Trnka R Bartuskova L Glogar P Kascakova N Kalman M . Are adolescent religious attendance/spirituality associated with family characteristics? Int J Environ Res Public Health. (2019) 16:1–16. doi: 10.3390/ijerph16162947, 31426330 PMC6721075

[51] Brassai L Piko BF Steger MF. Individual and parental factors related to meaning in life among Hungarian minority adolescents from Romania. Int J Psychol. (2013) 48:308–15. doi: 10.1080/00207594.2011.645483, 22376146

[52] Chen Y Kawachi I Berkman LF Trudel-Fitzgerald C Kubzansky LD. Does optimal parenting style help offspring maintain healthy weight into mid-life? Prev Med. (2019) 123:84–90. doi: 10.1016/j.ypmed.2019.03.001, 30844500 PMC6664443

[53] Gerhardt M Feng X Chan MHM. Long-term association of parental warmth with depression and obesity: mediation by conscientiousness. Health Psychol. (2021) 40:188–95. doi: 10.1037/hea0001061, 33630640

[54] Krause N. Longitudinal study of social support and meaning in life. Psychol Aging. (2007) 22:456–69. doi: 10.1037/0882-7974.22.3.456, 17874947

[55] Maccoby EE Martin JA. Socialization in the context of the family: parent–child interaction In: Mussen PH, editor. Handbook of child psychology: Vol. 4. Socialization, personality, and social development. New York: Wiley (1983). 1–101.

[56] Snyder CR Lopez SJ. Handbook of positive psychology. New York: Oxford university press (2001).

[57] Francis A Pai MS Badagabettu S. Psychological well-being and perceived parenting style among adolescents. Compr Child Adolesc Nurs. (2021) 44:134–43. doi: 10.1080/24694193.2020.1743796, 32302254

[58] Masten AS Coatsworth JD. The development of competence in favorable and unfavorable environments: lessons from research on successful children. Am Psychol. (1998) 53:205–20. doi: 10.1037/0003-066X.53.2.205, 9491748

[59] Hicks JA King LA. Positive mood and social relatedness as information about meaning in life. J Posit Psychol. (2009) 4:471–82. doi: 10.1080/17439760903271108

[60] Kearns SM Creaven AM. Individual differences in positive and negative emotion regulation: which strategies explain variability in loneliness? Personal Ment Health. (2017) 11:64–74. doi: 10.1002/pmh.1363, 27766765

[61] Ao M Luo D Hou H Li M Yang B. Association between latent profiles of parental parenting style and non-suicidal self-injury among Chinese adolescents: the mediating role of difficulties in emotion regulation. Chin J Clin Psychol. (2025) 33:254–60.

[62] Cheng L Xiao L Wang Y Gao R Liu X. The longitudinal impact of parenting styles on the mental health of gifted children: the mediating role of general self-efficacy. Chin J Clin Psychol. (2025) 33:770–6.

[63] Almeida TS Ribeiro O Freitas M Rubin KH Santos AJ. Loneliness and social functioning in adolescent peer victimization. Front Psychol. (2021) 12:664079. doi: 10.3389/fpsyg.2021.664079, 34276490 PMC8281116

[64] Heshmati S Blackard MB Beckmann B Chipidza W. Family relationships and adolescent loneliness: an application of social network analysis in family studies. J Fam Psychol. (2021) 35:182–91. doi: 10.1037/fam0000660, 33871279

[65] Yang Q. Zhang W. Wu H. Huang B. Zhang C. Niu G. . (2023). The association between perceived family financial stress and adolescent suicide ideation: a moderated mediation model. Behav. Sci., 13:948. 37998693 10.3390/bs13110948 PMC10669165

[66] Huang X. Hu N. Yao Z. Peng B. (2022). Family functioning and adolescent depression: A moderated mediation model of self-esteem and peer relationships. Front. Psychol. 13, 962147. 36017432 10.3389/fpsyg.2022.962147 PMC9396342

[67] Wang X. D. Wang X. L. Ma H. (1999). Handbook of mental health scales (Rev. ed.). Beijing: Chinese Journal of Mental Health Press.

[68] Jiang J. Lu Z. Jiang B. Xu Y. (2010). Revision of the short-form Egnahämn av Barndoms Uppfostran for Chinese. Psychological Development and Education, 26:99. doi:10.16187/j.cnki.issn1001-4918.2010.01.017

